# The Detection of Blood Pigments in the Fæces

**Published:** 1910-11-12

**Authors:** 


### Medicine.

### THE DETECTION OF BLOOD PIGMENTS IN THE FÆCES.

The detection of blood pigments in the fæces may be of the greatest value in diagnosing early malignant disease of the bowel, and in distinguishing between functional and organic disease of the stomach. When bleeding occurs from either the stomach or the duodenum the red blood corpuscles rapidly disintegrate, and the blood pigment is changed from hæmoglobin to hæmatin; hence the microscopical test for blood fails to detect blood in the fæces in the majority of cases in which its presence is not obvious to the naked eye. Even large quantities of hæmatin may only cause a slight darkening of the stool when mixed with any considerable quantity of solid fæces; and many food-stuffs and drugs may cause similar darkening, so that the mere colour of the stool is often deceptive.

### Some Useful Tests.

The following are some of the various tests that have been applied for the detection of minute quantities of blood pigment in the fæces:—

1. *Teichmann's Hæmin Test.*—It is said that hæmin crystals may be obtained from the fæces containing blood by the well-known medico-legal test that we owe to Teichmann. It is probable, however, that the amount of blood present must be more than a little before the hæmin crystals are obtainable.

2. *The Guaiacum Test.*—A quantity of fæces is thoroughly mixed with about one-third its volume of glacial acetic acid and thoroughly stirred; an equal volume of ether is then added and the whole shaken up and allowed to stand until it is thought that the ether has extracted the blood pigment. When the mixture is allowed to stand the ether floats to the top; about 2 cubic centimetres of the etherial extract are decanted off and the guaiacum and ozonic ether test applied to it in the ordinary way that is employed in testing urine. If blood pigment is present a deep greenish-blue colour is produced. To ensure sensitiveness the guaiacum solution should be freshly prepared.

3. *The Aloin Test.*—This is employed in the same way as the guaiacum test, aloin being substituted for guaiacum.

4. *The Benzidine Test.*—There are various modifications of the benzidine test, one of the most convenient perhaps being that described by Schlesinger and Holst. A piece of fæces the size of a pea is macerated in 5 cubic centimetres of water. The fæcal suspension is raised to boiling point and then cooled. The benzidine (Merck) is made up as a saturated solution in glacial acetic acid, 10 drops of this being mixed with 2 drops of the fæcal solution, and 3 drops of hydrogen peroxide are added. If blood pigment is present a deep green, or a deep blue colour is obtained within a few minutes.

5. *The Spectroscopic Test.*—The fæces are extracted with glacial acetic acid and ether as described above for the guaiacum test, and the etherial extract is examined spectroscopically in the ordinary way; the absorption bands of acid hæmatin are seen if blood pigment is present.

### Conclusions.

Dr. A. J. Clark (St. Bartholomew's Hospital Reports, vol. xlv.) has carried out numerous experiments with all the above tests, and has investigated the effects upon them of the administration by the mouth of various different food-stuffs and of various drugs. His summary of conclusions is as follows: (1) The guaiacum and benzidine tests will show the presence of the smallest quantities of blood in the fæces. (2) Of these tests the benzidine is the more convenient, but the guaiacum the more reliable. (3) The spectroscopic test when positive is of great value, but when negative it only proves the absence of large quantities of blood. (4) The smallest quantity of blood entering the bowel, either in the food, or by hæmorrhage, causes blood pigments to appear in the fæces. (5) For the above tests to be of clinical value the patient must have been for three days on a diet free from meat or green vegetables, or iodides. (6) To establish a negative result the fæces of three successive days must be tested. (7) When the above precautions are observed, a positive result indicates the occurrence of bleeding into the bowel. (8) Malignant disease of the stomach or intestine nearly always causes blood pigment to appear in the fæces. (9) Ulcer of the stomach or duodenum usually causes blood pigments to appear in the fæces. (10) The above tests may also be applied to test the presence of blood in the stomach contents, or in the urine.

